# Woven endobridge embolization versus microsurgical clipping for unruptured wide-neck cerebral aneurysms on middle cerebral artery bifurcation

**DOI:** 10.1186/s12883-025-04199-0

**Published:** 2025-05-08

**Authors:** Jun Hyeong Park, Hyun Jin Han, Jung-Jae Kim, Yong Bae Kim, Keun Young Park

**Affiliations:** Department of Neurosurgery, Severance Stroke Center, Severance Hospital, Yonsei University College of Medicine, 50 Yonsei-ro, Seodaemun-gu, Seoul, 03722 Republic of Korea

**Keywords:** Intracranial aneurysm, Middle cerebral artery, Woven endobridge, Clipping, Inverse probability of treatment weighting

## Abstract

Woven EndoBridge (WEB) is an endovascular treatment for wide-neck bifurcation aneurysms (WNBAs). This study aimed to clarify the efficacy and safety of the WEB comparing to clipping for treating the middle cerebral artery (MCA) WNBAs. For the matched analysis, we designed a retrospective, tertiary, single-center study including 288 MCA WNBAs treated with WEB (*n* = 37) or clipping (*n* = 251). Inverse probability of treatment weighting (IPTW) for the main analysis and 1:2 propensity score matching for the sensitivity analysis were used to adjust for aneurysm geometric parameters. Morbidity, major complications, and aneurysm occlusion grade were assessed at 1 year. In the IPTW analysis, no significant difference was observed in morbidity (2.1% vs. 1.6%, odds ratio [OR]: 1.34, *p* = 0.776) and major complications (3.3% vs. 3.1%, OR: 1.08, *p* = 0.926). Adequate aneurysm occlusion was achieved in 76.1% of the WEB group and 97.4% of the clipping group (OR: 0.09, *p* < 0.001). In line with the main analysis, the 1:2 propensity score matching analysis revealed no significant difference in the morbidity (OR: 2.00, 95% CI: 0.13–31.98, *p* = 0.624). This study supports that microsurgical clipping still requires to play a vital role, but WEB could be an alternative option for WNBAs in the MCA.

**Supplementary Information:**

The online version contains supplementary material available at 10.1186/s12883-025-04199-0.

## Introduction

Endovascular embolization is the primary treatment for unruptured cerebral aneurysms, and, it has expanded in scope along with endovascular devices and technique development in the past decade [1]. However, several concerns remain in treating middle cerebral artery (MCA) aneurysms. Aneurysms on MCA involve technical challenges because of a large portion of wide-necked aneurysms, incorporation of one or more M2 branch arteries, the existence of perforators on the aneurysm neck, and a relatively small parent artery size [2]. For these reasons, microsurgical clipping still plays a pivotal role in treating wide-neck bifurcation aneurysms (WNBAs) on MCA [3].

The Woven EndoBridge (WEB) device was introduced to treat WNBAs. Single-arm prospective studies have shown its safety and efficacy during short- to middle-term follow-up [4, 5, 6, 7]. Furthermore, several observational studies have suggested acceptable outcomes in comparison with conventional treatment modalities [8, 9]. However, there is limited information about the safety and efficacy, focusing on MCA WNBAs. Moreover, reports comparing with microsurgical clipping will be crucial to accept the safety and efficacy of WEB procedure.

In present study, we hypothesized that WEB is an alternative endovascular option in the direct comparison with microsurgical clipping for WNBAs on MCA. We designed an inverse probability of treatment weighting (IPTW) analysis using aneurysm geometric parameters to compare the clinical and radiologic outcomes at 1 year.

## Methods

### Patient population

**Fig. 1:**
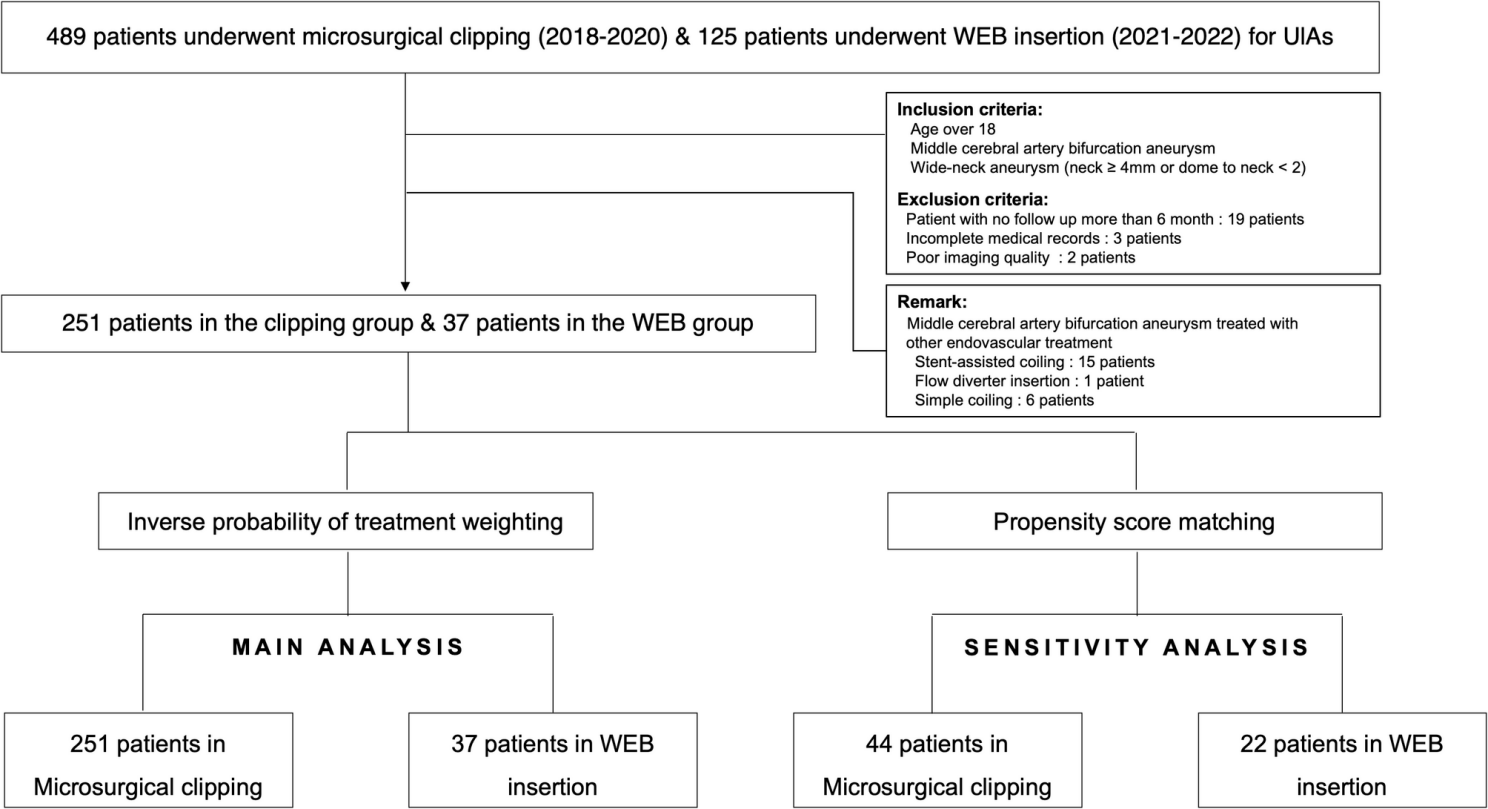
Flow chart. WEB: Woven EndoBridge, UIAs: unruptured intracranial aneurysms

The study protocol was reviewed and approved by the Institutional Review Board (IRB number: 4-2023-1677), and the need for patient consent for this study was waived owing to the study’s retrospective nature. However, informed consent for the procedure was obtained from all patients. The study was performed in accordance with the guidelines outlined by the Declaration of Helsinki, and the manuscript was written as per the Strengthening the Reporting of Observational Studies in Epidemiology (STROBE) guidelines. Between January 2018 and December 2020, 489 patients with unruptured intracranial aneurysms were treated via microsurgical clipping, and 125 patients were treated via WEB embolization between August 2021 (launch of WEB in the Republic of Korea) and December 2022 in a single tertiary hospital. This study only includes WNBAs on MCA defined as having a neck diameter ≥ 4 mm or a dome-to-neck ratio < 2. The exclusion criteria were ruptured aneurysm, recurrent aneurysm (previously treated via surgical or endovascular methods), lack of clinical or radiological follow-up, and inadequate imaging quality for analysis. This study analyzed a cohort of 288 individuals (37 and 251 treated with WEB and microsurgical clipping, respectively). Figure 1 shows a flowchart of the present study.

### Data collection

Patient demographics and aneurysm characteristics were reviewed retrospectively. Medical history including hypertension, diabetes mellitus, dyslipidemia, current smoking status, and alcohol consumption were collected. Before treatment, all aneurysms were evaluated using transfemoral catheter angiography (TFCA), and their geometric characteristics were obtained. Detailed information on the procedure, follow-up, and clinical and radiological outcomes were obtained from a prospectively collected aneurysm database.

### WEB procedure

A multidisciplinary team of neurosurgeons and neurointerventionalists made therapeutic decisions through a consensus. Before the procedure, each patient was prescribed dual antiplatelet therapy consisting of aspirin (100 mg once daily) and clopidogrel (75 mg once daily) for at least five days. Platelet function tests were performed using the VerifyNow P2Y12 Assay (Accumetrics, San Diego, California, USA) to measure P2Y12 reaction units (PRU). Patients with a PRU > 220 were classified as clopidogrel hyporesponders and administered additional cilostazol (100 mg daily). For patients without prior antiplatelet medication, a loading dose of aspirin (300 mg) and clopidogrel (300 mg) was administered either one day before or on the day of the procedure.

All procedures were conducted under general anesthesia and were routinely performed using a transfemoral approach. After placement of a 6–7 F shuttle sheath (Cook Medical, Bloomington, IN, USA) in the cervical segment of the relevant parent artery, a 5–6 F intermediate catheter (SOFIA; Microvention, Tustin, CA, USA) was coaxially introduced into the shuttle sheath as far distally as possible. The WEB device was delivered through a Headway 17 or a VIA microcatheter (Microvention). The appropriate WEB size was selected according to the + 1/-1 rule (1 mm larger than the average dome size and 1 mm smaller than the lowest aneurysm height). For further adjustment, we used preprocedural virtual simulation. If there was a large discrepancy between two selection methods, we principally followed the + 1/-1 rule. Adjunctive treatments, such as balloon or stent placement, were performed at the operator’s discretion, as deemed necessary. After deploying the WEB device, a flat-panel detector computed tomography (Vaso-CT) was performed before detachment, and adequate coverage or parent artery patency was evaluated. After the procedure, aspirin was continued until the first outpatient visit (1 month after discharge). For patients requiring additional stents, dual antiplatelet therapy was prescribed for 6 months, followed by aspirin monotherapy.

### Microsurgical clipping

Microsurgical clipping was performed using the pterional or composite approach with the assistance of an operating microscope (OPMI R PENTEROR 800, Carl Zeiss AG, Oberkochen, Germany). Somatosensory and motor-evoked potential monitoring was performed during the operation. In addition, we evaluated the parent artery patency and aneurysm occlusion using intraoperative Doppler ultrasonography and indocyanine green video angiography. The contralateral side of the aneurysm that was not visible under the microscope was examined using an operative endoscope (Arthrex, Munich, Germany).

### Follow-up protocol

All the participants followed an institutionally standardized protocol. Patients treated with WEB were followed up at 1, 6, and 12 months. Radiographic evaluation was conducted at 6 months using magnetic resonance angiography (MRA) and at 12 months using TFCA or MRA. For patients who underwent microsurgical clipping, clinical follow-up was conducted at 1, 6, and 12 months. Radiological follow-up was performed using computed tomography angiography (CTA) at 5 days and 12 months postoperatively.

### Clinical and radiologic outcomes

Clinical outcomes were evaluated using the modified Rankin Scale (mRS) [10]. Morbidity was defined as an mRS score > 1 when the preoperative mRS score was ≤ 1, and as an increase of 1 point when preoperative mRS score was > 1. Considering that treating unruptured aneurysms is mostly elective for non-symptomatic patients, the criteria for morbidity were lowered and strictly verified. Radiological outcomes were assessed based on the adequate occlusion rate at the last follow-up. Occlusion grade was evaluated using WEB occlusion scale (WOS) [11] in the WEB group and Sindou grading scale [12] in the clipping group. Adequate occlusion was defined as WOS grades A, B and C in the WEB group and Sindou grade I, and II in the clipping group.

We recorded all complications during the follow-up period. Major complications were defined as events that led to permanent disability or conditions requiring additional surgery or hospital admission, such as relevant territory infarction, intracranial hemorrhage, wound infection, and mortality. Symptoms that resolved spontaneously or with conservative care without discharge delay were categorized as minor complications.

### Statistical analysis

Categorical variables were reported as frequencies, and comparative analysis was performed using chi-squared and Fisher’s exact tests. Continuous variables were described as mean and standard deviation (SD), with comparisons made using the t-test and linear regression. To control for potential selection bias, IPTW was implemented. The treatment weighting score was computed with a logistic regression model using preprocedural baseline characteristics including the patient sex, age, aneurysm width, height, and neck size for matching covariates. The Rao-Scott chi-squared test, weighted logistic regression and weighted linear regression were used for categorical and continuous variables in adjusted analysis.

Propensity score matching (PSM) was used for sensitivity analysis. Propensity scores, calculated using the same variables as in the IPTW model, were used for matched comparisons of outcomes with a 1:2 nearest-neighbor matching algorithm. After matching, linear mixed model, generalized estimating equations, and conditional logistic regression were used for comparative analysis. All the statistical analyses were performed using Statistical Analysis Software (version 9.4, SAS Inc., Cary, NC, USA). Adjusted comparisons are presented as odds ratios (OR) with 95% confidence intervals (CI), and statistical significance was defined as *p* < 0.05.

## Results

### Baseline characteristics

Table 1 shows the patients’ baseline characteristics. Participants’ mean age was 62.0 years (SD: 8.9) and 60.1 years (SD: 8.0) in the WEB and clipping groups, respectively (*P* = 0.195). No significant differences in demographics or medical history were found between the two treatment groups, except for aneurysm morphology. The WEB group had a significantly greater aneurysm height (4.76 vs. 3.56 mm, *P* < 0.001) and aspect ratio (1.26 vs. 0.97, *P* < 0.001).

**Table 1:** Baseline characteristics of study participants with unruptured wide-neck bifurcation aneurysm on the middle cerebral artery

	**Before IPTW**				After IPTW			
	**WEB**	Clipping	*p*-value	SMD	**WEB**	Clipping	*p*-value	SMD
	(N = 37)	(N = 251)			(N = 37)	(N = 251)		
**Age**, **yrs**	62.0 ± 8.9	60.1 ± 8.0	0.195	0.22	59.6 ± 1.5	60.3 ± 0.5	0.712	0.072
**Male (%)**	12 (32.4)	65 (25.9)	0.402	0.144	12 (28.5)	65 (26.8)	0.861	0.037
**BMI**	24.8 ± 3.3	24.2 ± 2.9	0.311	0.172	25.6 ± 0.6	24.3 ± 0.2	0.149	0.329
**Comorbidities (%)**								
Hypertension	21 (56.8)	136 (54.2)	0.769	0.052	21 (61.4)	136 (53.9)	0.467	0.153
Diabetes mellitus	10 (27.0)	51 (20.3)	0.351	0.158	10 (26.3)	51 (20.5)	0.517	0.137
Hyperlipidemia	18 (48.7)	146 (58.2)	0.275	0.192	18 (54.4)	146 (58.5)	0.698	0.082
Smoker	4 (10.8)	43 (17.1)	0.331	0.183	4 (6.8)	43 (17.0)	0.081	0.321
Alcohol consumption	12 (32.4)	55 (21.9)	0.157	0.238	12 (36.9)	55 (22.3)	0.113	0.323
**Aneurysm Morphology**								
Width (mm)	4.94 ± 1.73	4.75 ± 2.16	0.616	0.095	4.61 ± 0.24	4.79 ± 0.14	0.54	0.087
Height (mm)	4.76 ± 1.86	3.56 ± 1.90	<.001*	0.639	3.90 ± 0.26	3.72 ± 0.13	0.577	0.087
Neck diameter (mm)	3.81 ± 1.31	3.71 ± 1.41	0.673	0.076	3.58 ± 0.20	3.73 ± 0.09	0.593	0.094
Aspect ratio	1.26 ± 0.33	0.97 ± 0.34	<.001*	0.872	1.10 ± 0.05	1.01 ± 0.02	0.105	0.254
Dome to Neck ratio	1.32 ± 0.23	1.29 ± 0.30	0.532	0.12	1.32 ± 0.04	1.29 ± 0.02	0.506	0.113

IPTW: inverse probability of treatment weighting, WEB: woven endobridge, SMD: standardized mean difference, SD: standard deviation, BMI: body mass index. Values are presented as the number and proportion (%) of patients unless otherwise specified. Asterisk indicates statistical significance (*p* < 0.05)

### Clinical and radiologic outcomes before IPTW

The mean clinical follow-up durations in the WEB and clipping groups were 299.8 (SD: 92.0) and 320.4 (SD: 93.5) days, respectively (*P* = 0.21). In the WEB group, 2 patients (5.4%) had major adverse events. One patient (2.7%) had intraoperative aneurysm rupture and fatal subarachnoid hemorrhage resulting in mortality. The other patient experienced left-side weakness from an ischemic stroke after WEB with adjuvant stent insertion but showed symptom improvement with an mRS score of 1 at the last follow-up. Major adverse events in the clipping group occurred in 8 (3.2%) patients. The leading cause of major adverse events were ischemic stroke after operation (*n* = 6); 4 (1.6%) patients ended with unfavorable functional outcome with mRS score over 1 (mRS 2, *n* = 2; mRS 3, *n* = 2), the other 2 patients recovered from symptoms at 1 year follow up. Following cause of major adverse events were a case of wound infection, which required revision surgery, and a case of postoperative cognitive decline (mRS 1). There was no mortality case in the clipping group. Details of the outcomes and the adverse events are presented in Tables 2 and 3.

**Table 2:** Clinical and radiologic outcome before and after inverse probability of treatment weighting

	Before IPTW				After IPTW			
	WEB	Clipping	OR (95% CI)	*p*-value	WEB	Clipping	OR (95% CI)	*p*-value
	(N = 37)	(N = 251)			(N = 37)	(N = 251)		
**Clinical Follow-up**								
Interval (days, Mean ± SD)	299.8 ± 92.0	320.4 ± 93.5		0.212	298.9 ± 12.6	322.5 ± 6.0		0.093
Morbidity (%)	1 (2.7)	4 (1.6)	1.72 (0.19–15.78)	0.634	1 (2.1)	4 (1.6)	1.34 (0.18–10.32)	0.776
Major Complications (%)	2 (5.4)	8 (3.2)	1.74 (0.35–8.51)	0.497	2 (3.3)	8 (3.1)	1.08 (0.22–5.32)	0.926
**Radiologic Follow-up**								
Interval (days, Mean ± SD)	301.6 ± 104.3	300.9 ± 173.6		0.976	304.13 ± 22.11	303.69 ± 10.47		0.986
Adequate occlusion (%)	27 (75.0)	244 (97.2)	0.09 (0.03–0.25)	<.0001*	27 (76.1)	244 (97.4)	0.09 (0.03–0.23)	<.0001*

IPTW: inverse probability of treatment weighting, WEB: woven endobridge, OR: odds ratio, CI: confidence interval, SD: standard deviation, mRS: modified Rankin scale. Values are presented as the number and proportion (%) of patients unless otherwise specified. Asterisk indicates statistical significance (*p* < 0.05)

**Table 3:** Details of complications of the WEB and clipping groups

	WEB	Clipping
	(N = 37)	(N = 251)
**Overall adverse events (%)**	2 (5.4)	8 (3.2)
**Acute Ischemic stroke**	1 (2.7)	6 (2.4)
**Hemorrhagic complication**		
Subarachnoid hemorrhage	1 (2.7)	0
Subdural/Epidural hemorrhage	-	0
Wound infection	-	1 (0.4)
Postoperative cognitive decline	-	1 (0.4)
**Mortality (%)**	1 (2.7)	0

WEB: woven endobridge. Values are presented as number and proportion (%) of the patients not otherwise specified

**Fig. 2:**
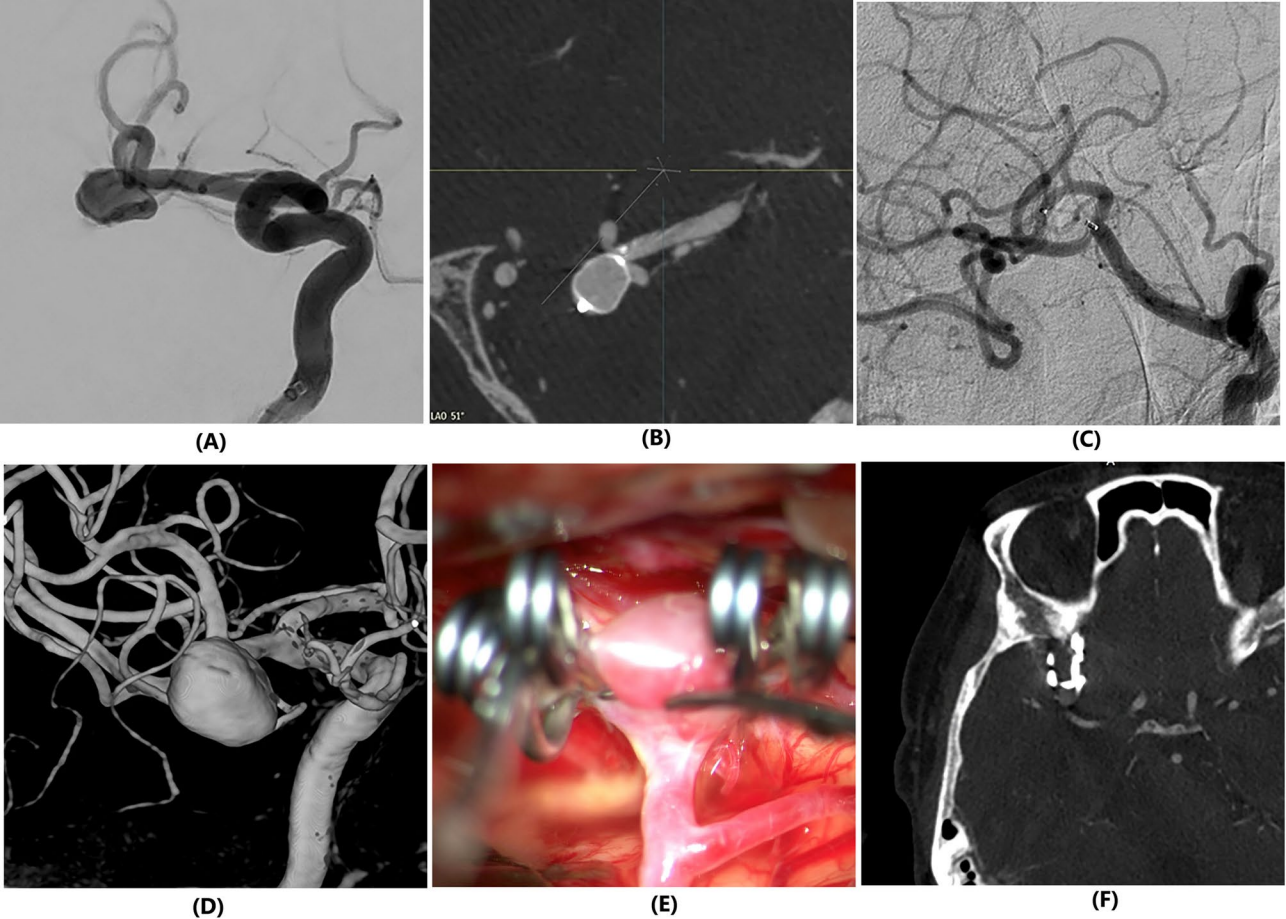
Case demonstration. Two-dimensional (2-D) digital subtraction angiography (DSA) showing a middle cerebral artery aneurysm planned for treatment with Woven EndoBridge (WEB) device (**A**). Intra-procedural flat-panel detector computed tomography image demonstrating placement of the WEB device (**B**). Twelve-month follow up 2-D DSA showing complete occlusion of aneurysm (**C**). Three-dimensional DSA showing a middle cerebral artery aneurysm planned for microsurgical clipping (**D**). Intraoperative view of aneurysm clipping (**E**). Twelve-month follow-up computed tomography angiography demonstrating a residual neck remnant (**F**)

Radiological follow-up was performed for 36 patients in the WEB group (97.3%, mean 301.6 days, SD: 104.3). Adequate occlusion (WOS A, B and C) was achieved in 27 patients (75.0%). In the clipping group, radiologic follow-up was available for 246 patients (98.0%, mean 300.9 days, SD: 173.6), and adequate occlusion (Sindou I and II) was noted in 244 patients (97.2%). Figure 2 illustrates representative cases from each group. No case required retreatment in both groups.

### Clinical and radiologic outcomes after IPTW

After IPTW, baseline characteristics, which were included in calculation of propensity score, were well balanced. Rate of morbidity did not differ between the WEB and clipping groups (2.1% vs. 1.6%; OR:1.34; 95% CI: 0.18–10.32, *p* = 0.776). In addition, no differences were noted in major complications (3.3% vs. 3.1%; OR: 1.08; 95% CI: 0.22–5.32, *p* = 0.926). Regarding radiological outcomes, adequate occlusion rate was significantly lower in WEB group (76.1% vs. 97.4%; OR: 0.09; 95% CI: 0.03–0.23, *p* < 0.001).

### Sensitivity analysis with PSM

We selected 36 and 72 patients from the WEB and clipping groups, respectively, using propensity score matching. Aneurysm characters and baseline demographics in both groups were well balanced after PSM except for dome-to-neck ratio (1.33 vs. 1.24, *P* = 0.035, Supplementary Table 1). No significant difference was observed in the morbidity rate (2.8% vs. 1.4%, OR: 2.00, 95% CI: 0.13–31.98, *p* = 0.624) and major complications between both groups (5.6% vs. 1.4%, OR: 4.00, 95% CI: 0.36–44.11, *p* = 0.258). Align with main analysis of IPTW, rate of adequate occlusion was significantly lower in WEB group (74.3% vs. 98.6%, OR: 0.06, 95% CI: 0.01–0.44, *p* = 0.006). Details are provided in Supplementary Table 2.

## Discussion

This study compared the clinical and radiological outcomes of WEB embolization and microsurgical clipping of WNBAs on MCA. In the IPTW analysis, WEB embolization was comparable to microsurgical clipping for treating WNBAs on MCA with comparable functional outcomes (2.1% vs. 1.6%; OR:1.34; 95% CI: 0.18–10.32, *p* = 0.776) and complication rates (3.3% vs. 3.1%; OR: 1.08; 95% CI: 0.22–5.32, *p* = 0.926). Radiologic outcomes showed an acceptable occlusion rate for the WEB group (76.1%); however, microsurgical clipping showed a significantly higher occlusion rate (97.4%; OR: 0.09; 95% CI: 0.03–0.23, *p* < 0.001). Taken together, our investigators suggest that microsurgical clipping was still a viable option for WNAs on MCA. However, WEB embolization had natural advantage compared with microsurgical clipping, generally shorter procedure and anesthesia time and less invasiveness. Therefore, we suggested that WEB embolization could be an endovascular option for the patients with unsuitable for microsurgical clipping.

Historically, treating wide-neck aneurysms has been a significant challenge for endovascular therapy [13]. In this reason, the adjunctive technique or devices, balloon- or stent-assisted coiling, or flow diverters were introduced, and recent reports showed the acceptable outcomes for wide-neck aneurysms. A meta-analysis of open-cell stent-assisted coiling showed that the subgroup of wide-neck aneurysms had a 93% adequate occlusion rate at the 12-month follow-up with an overall complication rate of 6% [14]. However, aneurysms on MCA have additional considerations: a long distance to target aneurysms, incorporation of another M2 division, and numerous lenticulostriate arteries originating from M1 or proximal M2 [2, 15]. These obstacles made neurointerventionalists require more discreet planning and advanced techniques. Despite technical improvements and device development, the outcome of endovascular treatment for WNBAs in the MCA remains relatively insufficient, with lower occlusion rates and a higher risk of recanalization [13, 16, 17].

The WEB device was introduced as an intrasaccular flow disruption device to isolate the aneurysm from normal circulation. A consistent metal coverage of 35–45% increased resistance at the level of aneurysm ostium, promoting intra-aneurysmal thrombosis and providing a scaffold for neointima bridging [18]. The braided, self-expanding design sustained an appropriate radial force against the aneurysm wall to promote device adaptation and secure device position, even in a wide neck [19]. In clinical studies, WEBCAST, WEBCAST-2, and the French Observatory have reported overall complete occlusion rates of 52.9% and neck remnant rates of 26.1% in a single-arm fashion [4, 5, 6, 7]. A large multicenter study, including a subgroup of 206 MCA bifurcation aneurysms, showed a promising adequate occlusion rate of 78.6% [20]. Despite the promising efficacy and safety outcomes reported in previous single-arm studies, there is still a scarcity of information comparing this new treatment to pre-existing modalities. Therefore, we designed a matched comparative study, which demonstrated similar and acceptable clinical and radiologic outcomes, supporting WEB implantation as an alternative to microsurgical clipping for WNBAs in the MCA.

Functional outcomes were comparable between the WEB and clipping groups in the regard of morbidity rate and major complications. Safety profiles were consistent with previous reports. Pierot et al., integrating data from three major pioneering prospective studies, reported a 3.0% global morbidity rate at 1 month (1.2% procedure- and 1.8% disease-related) and a 7.8% symptomatic thromboembolic complication rate at 1 year after WEB device implantation [7]. In a large series of 266 WEB-treated aneurysms, a total of 35 cases (13.0%) of thromboembolic events were noted. Notably, a high proportion of these events occurred intraoperatively (23 cases, 65.7%), as observed in our case series [21]. Clipping related morbidities were observed in 4 cases, composed of thromboembolic complications. Morbidity rate reported in our case series is comparable to the 6.7% reported in large meta-analysis, Kotowski et al. [22]. However, revision surgery after wound infection, and significant cognitive decline were also reported as major complication. This underscores risk of craniotomy, such as postoperative cognitive decline and large skin wound, compared to endovascular therapy [23].

The angiographic results of the WEB group in our cohort showed an adequate occlusion rate similar with those in prospective pilot studies, although significantly lower compared to the clipping group. Nevertheless, several factors need to be considered to interpret this disparity. The follow-up angiographic modalities differed between the two groups, with a higher proportion of TFCA in the WEB group (WEB: 62.2% for TFCA, clip: 87.0% for CTA). The occlusion rate may appear much lower when assessed via 3-dimensional rotational digital subtraction angiography due to its higher spatial resolution and resistance to metal artifacts [24]. Another concern is the higher rate of recanalization and retreatment. However, in this study, no retreatments were performed in the WEB group. Among nine cases with aneurysmal remnants, seven showed minor recanalization and two showed intra-device contrast filling, indicating isolated intra-device flow with partial thrombosis. This type of intra-device contrast filling may represent an ongoing process of aneurysm obliteration, and a previous study described the disappearance of intra-device contrast filling in 8 out of 9 patients without further intervention [25]. Thus, a 1-year follow-up might have been insufficient for cases with intra-device contrast filling, and a long-term follow-up study will be essential, with an expected serial increase in the occlusion rate in the WEB group [26].

The primary limitations of this study include its retrospective design and the fair size of the patient group, leading to incomplete datasets, selection bias, and unidentified confounders. These aspects, along with the study’s single-center nature, constrain our findings generalizability. Moreover, omitting variables that could potentially affect outcomes, such as the length of the M1 segment, the presence of daughter sacs, and the tilting angle of aneurysms, may limit the comprehensiveness of our analysis. Discrepancies in the durations of follow-up period, imaging techniques, and group sizes between the two treatment modalities may have influenced the results. Our study evaluated radiologic results at one year, but we were unable to assess the rates of retreatment and mid- or long-term occlusion. Consequently, it was strongly required to compare the WEB and clipping groups using a variety of outcome measures and long-term follow-up outcomes. Lastly, including early cases in the WEB group when the device was newly introduced may have led to a disparity in surgical expertise, favoring the clipping group. Despite these limitations, our study employed PSM to minimize bias and enhance statistical comprehensiveness. In addition, by focusing specifically on MCA aneurysms, our research offers detailed guidance for clinical decision-making.

## Conclusion

This study demonstrated the modest efficacy and safety of the WEB in the comparison with microsurgical clipping. Therefore, microsurgical clipping still requires to play a vital role, but WEB could be an alternative option for WNBAs in the MCA.

## Electronic supplementary material

Below is the link to the electronic supplementary material.

Supplementary Material 1

## Data Availability

The data supporting the findings in this study can be obtained from the corresponding author upon reasonable request.

## Abbreviations

CI: Confidence interval
CTA: Computed tomography angiography
IPTW: Inverse probability of treatment weighting
MCA: Middle cerebral artery
MRA: Magnetic resonance angiography
mRS: Modified Rankin scale
OR: Odds ratio
PRU: P2Y12 reaction unit
PSM: Propensity score matching
SD: Standard deviation
TFCA: Transfemoral catheter angiography
WEB: Woven EndoBridge
WNBA: Wide-neck bifurcation aneurysm
