# Supplementary material for: Woven endobridge embolization versus microsurgical clipping for unruptured wide-neck cerebral aneurysms on middle cerebral artery bifurcation
Source: BMC Neurol. 2025 May 8;25:202. doi: 10.1186/s12883-025-04199-0 (PMC12060329; doi:10.1186/s12883-025-04199-0)
Supplement: Supplementary file 1 — Supplementary Material 1 [file 12883_2025_4199_MOESM1_ESM.pdf]

## Supplementary Materials

**Supplementary Table 1. Baseline characteristics of the WEB and clipping group after propensity score matching.** WEB: Woven EndoBridge, SMD: standardized mean difference, SD: standard deviation, BMI: body mass index. Values are presented as number and proportion (%) of the patients not otherwise specified. Asterisk indicates statistical significance (p < .05).

| | **WEB (N = 36)** | **Clipping (N = 72)** | ***p*-value** | **SMD** |
|---|---|---|---|---|
| **Age, yrs** | 61.5±8.6 | 61.5±8.9 | 0.999 | 0.001 |
| **Male (%)** | 12 (33.3) | 20 (27.8) | 0.547 | 0.121 |
| **BMI** | 24.8±3.3 | 24.6±2.7 | 0.724 | 0.071 |
| **Comorbidities** | | | | |
| Hypertension | 20 (55.6) | 38 (52.8) | 0.788 | 0.056 |
| Diabetes mellitus | 10 (27.8) | 15 (20.8) | 0.355 | 0.162 |
| Hyperlipidemia | 17 (47.2) | 42 (58.3) | 0.288 | 0.224 |
| Smoker | 4 (11.1) | 13 (18.1) | 0.353 | 0.198 |
| Alcohol consumption | 12 (33.3) | 19 (26.4) | 0.408 | 0.152 |
| **Aneurysm Morphology** | | | | |
| Width (mm) | 4.92±1.75 | 4.74±2.64 | 0.676 | 0.082 |
| Height (mm) | 4.64±1.74 | 4.53±2.38 | 0.739 | 0.055 |
| Neck diameter (mm) | 3.77±1.30 | 3.83±1.67 | 0.837 | 0.039 |
| Aspect ratio | 1.25±0.32 | 1.20±0.36 | 0.182 | 0.144 |
| Dome to neck ratio | 1.33±0.23 | 1.24±0.32 | 0.035* | 0.329 |

**Supplementary Table 2. Clinical and radiologic outcome after propensity score matching.** WEB: Woven EndoBridge, OR: odds ratio, CI: confidence interval, SD: standard deviation, mRS: modified Rankin Scale. Values are presented as number and proportion (%) of the patients not otherwise specified. Asterisk indicates statistical significance (p < .05).

| | WEB (N = 36) | Clipping (N = 72) | OR (95% CI) | *p*-value |
|---|---|---|---|---|
| **Clinical Follow-up** | | | | |
| Interval (days, Mean±SD) | 297.7±92.4 | 328.2±88.4 | | 0.092 |
| Morbidity (%) | 1 (2.8) | 1 (1.4) | 2.00 (0.13-31.98) | 0.624 |
| Major Complications (%) | 2 (5.6) | 1 (1.4) | 4.00 (0.36-44.11) | 0.258 |
| **Radiologic Follow-up** | | | | |
| Interval (days, Mean±SD) | 299.8±105.3 | 305.9±163.7 | | 0.839 |
| Adequate occlusion (%) | 26 (74.3) | 71 (98.6) | 0.06 (0.01-0.44) | 0.006* |
